# One stone for multiple birds: PigmR integrates multiple defense pathways for high and broad-spectrum blast resistance in rice

**DOI:** 10.1007/s44154-025-00228-7

**Published:** 2025-03-31

**Authors:** Zhuoer Xie, Leiyun Yang, Zhengguang Zhang

**Affiliations:** 1 State Key Laboratory of Agricultural and Forestry Biosecurity, College of Plant Protection, Nanjing Agricultural University, Nanjing, 210095 China; 2 Key Laboratory of Integrated Management of Crop Diseases and Pests, Ministry of Education, Nanjing, 210095 China; 3 Key Laboratory of Plant Immunity, Nanjing Agricultural University, Nanjing, 210095 China

## Main text

Crop pathogens pose a significant challenge to agricultural productivity and remain a persistent threat to global food security. Engineering the plant immune system offers a promising genetic solution to combat crop diseases caused by a wide arrange of agriculturally relevant pathogens. Plant immune response is primarily initiated by plasma membrane (PM)-localized pattern recognition receptors (PRRs) and intracellular nucleotide-binding leucine-rich repeat proteins (NLRs). PRRs detect conserved microbial features, such as fungal chitin and bacterial flagellin, to activate pattern-triggered immunity (PTI), which helps defend against pathogen invasion. However, pathogens can counteract PTI by secreting effector proteins into plant cells to suppress immune responses and facilitate infection. In response, NLRs recognize these effector proteins and activate effector-triggered immunity (ETI) to halt pathogen proliferation. PTI and ETI function synergistically to ensure a robust immune response (Ngou et al. 2021; Yuan et al. 2021). Incorporating PTI and ETI components, such as resistance genes, into crop varieties has proven to be one of the most effective and sustainable approaches for mitigating crop diseases.

**Fig. 1:**
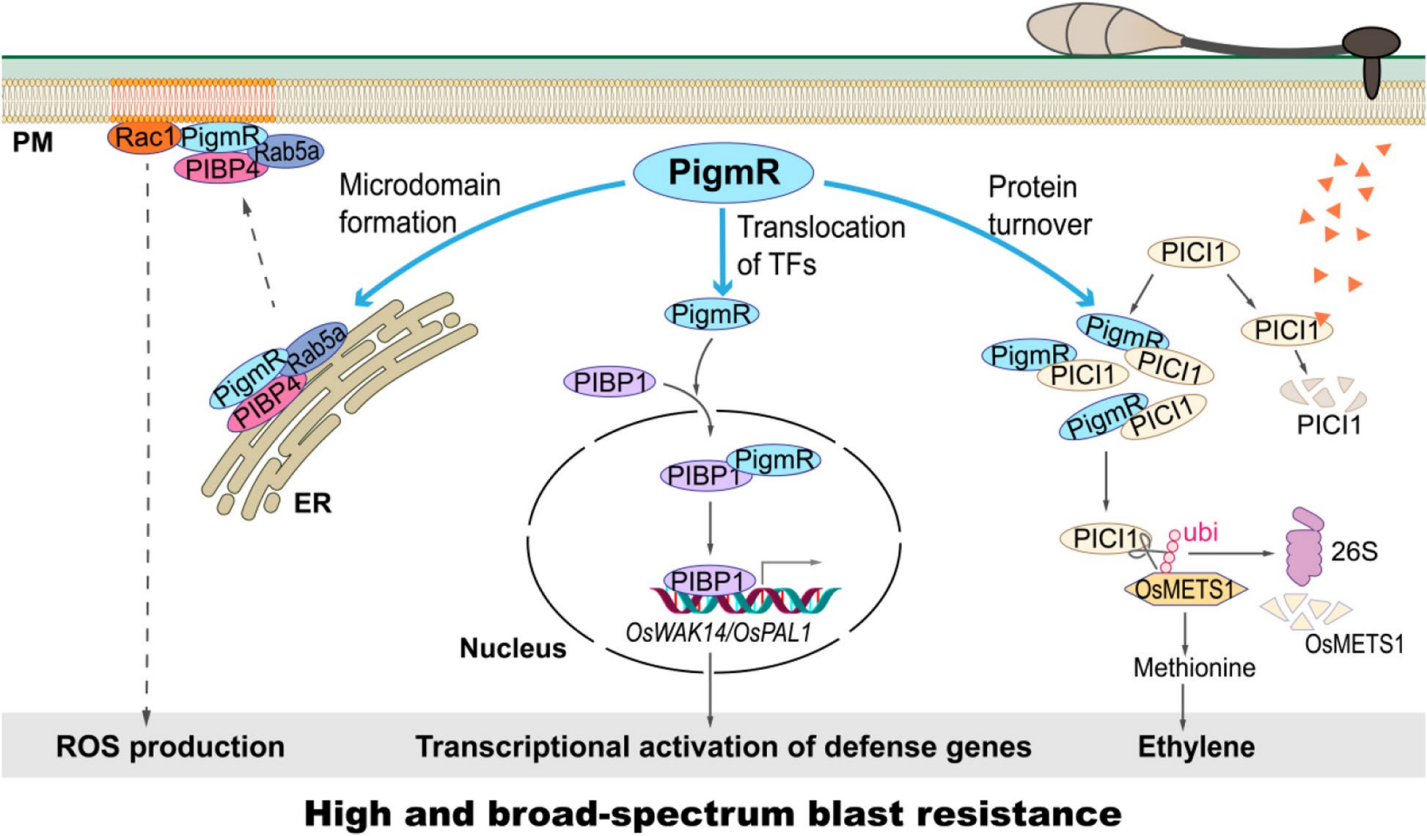
PigmR plays a multifaceted role in blast resistance. 1) PigmR is translocated by PIBP4-Rab5a module into plasma membrane (PM), where it forms microdomains essential for its interaction with Rac1, thereby promoting ROS production. 2) PigmR facilitates the translocation of the transcription factor PIBP1 into the nucleus for transcriptional activation of defense genes, such as *OsWAK14* and *OsPAL1*. 3) The methionine synthetase OsMETS1 mediates methionine and subsequent ethylene biosynthesis, which enhances blast resistance. However, OsMETS1 is subject to degradation by the 26S proteasome. PICI1 stabilizes OsMETS1 by removing ubiquitin modifications, thereby promoting blast resistance. On the other hand, PICI1 is targeted for degradation by effectors, which is inhibited by PigmR through competitive binding to PICI1

*Magnaporthe oryzae* (*M. oryzae)* causes rice blast disease and severely threatens global food security. To date, at least 30 blast resistance genes have been identified (Yin et al. 2021), with most being NLR genes originating from wild rice species or landraces. NLRs often confer race-specific blast resistance, which limits their application in plant breeding. The rice variety Gumei 4 (GM4), known for its durable and broad-spectrum blast resistance to multiple *M. oryzae* isolates, has been extensively utilized as a donor in breeding blast resistant rice varieties for over 50 years (Deng et al. 2017). The gene conferring this resistance was mapped to the *Pigm* resistance locus (Deng et al. 2006). Near-isogenic lines harboring *Pigm* (NIL-*Pigm*) in the Nipponbare background exhibit high and broad-spectrum resistance to rice blast. Despite its significance, the specific gene responsible for resistance within the *Pigm* locus remained unidentified until 2017, when Deng and colleagues isolated the disease resistance gene *PigmR* (*Pigm*, *Resistant*) (Deng et al. 2017), resolving a long-standing mystery in the field. Subsequent research on *PigmR* has led to remarkable and exciting advances in understanding various defense pathways, including transcriptional activation of defense genes, the interplay between PTI and ETI, and the role of microdomain at the PM. These findings highlight PigmR as a multifaceted player in plant immunity, serving as a “one stone for multiple birds” strategy (Fig. 1).

## The stone: the rice blast resistance gene *PigmR*

*PigmR* resides in an NLR gene cluster and encodes a coiled-coil (CC) type NLR (CNL) protein (Deng et al. 2017). Introducing *PigmR*, but not other NLR genes within this cluster, into Nipponbare confers high and broad-spectrum resistance to *M. oryzae*, mirroring the resistance observed in the donor GM4. Interestingly, *PigmS* (*Pigm*, *Susceptible*), a neighboring CNL gene of *PigmR*, antagonizes *PigmR*-mediated immunity (Deng et al. 2017). Ectopic expression of *PigmS* in NIL-*Pigm* or *PigmR* transgenic plants enhances susceptibility to *M. oryzae* infection. Further studies revealed that PigmR forms homodimers to mediate disease resistance, whereas PigmS interacts with PigmR to form heterodimers, thereby inhibiting PigmR homodimerization and attenuating its immune function. Additionally, *PigmR* is constitutively expressed across all tissues, ensuring strong disease resistance but at the cost of reduced yield. In contrast, *PigmS* is predominantly expressed in pollen, and *PigmS* transgenic plants exhibit increased seed setting and higher grain yield. The concurrent expression of *PigmR* and *PigmS* in NIL-*Pigm* lines maintains a defense-yield balance. NIL-*Pigm* lines exhibit no yield penalty under non-pathogenic conditions, whereas they substantially improved yield under pathogenic conditions. This breakthrough not only uncovers a new mechanism by which defense and yield could be balanced by antagonistic NLRs, but also delineates the genetic basis for breeding blast resistant rice varieties using GM4 or *Pigm*.

## Bird one: the PigmR-PIBP1-WAK14/PAL1 transcriptional regulatory cascade

To elucidate the molecular mechanism underlying PigmR-mediated blast resistance, Zhai and colleagues conducted a yeast-two hybrid (Y2H) screen using PigmR CC domain as the bait against a rice cDNA library, and identified PIBP1 (PigmR-Interacting and Blast resistance Protein 1) as a PigmR interactor (Zhai et al. 2019). PIBP1 encodes a putative transcription factor. Combinatory analyses of chromatin immunoprecipitation sequencing and RNA-sequencing identified two previously reported positive regulators of blast resistance, *OsWAK14* (*Wall-Associated Kinase 14*) and *OsPAL1* (*Phenylalanine Ammonia Lyase 1*) (Delteil et al. 2016; Zhou et al. 2018), as potential direct targets of *PIBP1*. Further analyses validated that PIBP1 directly binds to the promoter regions of *OsWAK14* and *OsPAL1*, activating their expression and thereby enhancing rice blast resistance. Of note, PIBP1-YFP was localized to the nucleus in 95.3% of NIL-*Pigm* protoplasts, compared to only 59.7% in Nipponbare protoplasts, suggesting that PigmR facilitates PIBP1 nuclear translocation. This study revealed a PigmR-PIBP1-WAK14/PAL1 regulatory cascade in rice blast resistance, underscoring the critical role of PigmR in the transcriptional regulation of defense genes.

## Bird two: the PigmR-PICI1-OsMETS1-ethylene metabolic cascade

When integrated the PigmR interactome with chitin-induced proteins, Zhai and colleagues identified three PigmR-interacting and chitin-induced proteins (PICIs) (Zhai et al. 2022). Among these, PICI1 is a putative deubiquitinase. Further analysis of the PICI1 interactome and chitin-induced ubiquitome identified the methionine synthase OsMETS1 as a potential direct substrate of PICI1. Deubiquitination assays validated that PICI1 stabilizes OsMETS1 via deubiquitination. Loss of *OsMETS1* or *PICI1* in NIL-*Pigm* reduces methionine content and decreases resistance to both virulent and avirulent strains compared with NIL-*Pigm*, and these phenotypes can be rescued by exogenous methionine supplement. As methionine is the precursor for ethylene biosynthesis, PTI-induced ethylene accumulation is as expected reduced in *OsMETS1*-KO and *PICI1*-KO plants. Treatment with the ethylene precursor ACC rescues *OsMETS*-RNAi and *PICI1*-KO resistance to the virulent strain TM21, and treatment with the ethylene biosynthesis inhibitor AVG compromises NIL-*Pigm* resistance to the avirulent strain TH12. These findings demonstrate a critical role of the PICI1-OsMETS1-ethylene metabolic cascade in regulating PTI and ETI.

Interestingly, PICI1 can be targeted for degradation by multiple avirulent effectors, such as AvrPi9, suggesting that PICI1 might be an immune hub that could be hijacked by various pathogen effectors. Of note, PigmR protects PICI1 from effectors by its competitive binding to PICI1, rebooting the PICI1-OsMETS1-ethylene immune signaling. This study showcases a new PigmR-PICI1-OsMETS1-ethylene metabolic cascade that integrates both PTI and ETI, ensuring broad-spectrum resistance against rice blast disease.

## Bird three: PIBP4/Rab5a-PigmR-Rac1 mediated microdomain formation for ROS production

In addition to PIBP1, PIBP4 was also identified as a PigmR interactor in the Y2H screen, and it interacts with PigmR at the endoplasmic reticulum (ER) and PM (Liang et al. 2024). PIBP4 is a putative PRENYLATED RAB ACCEPTOR domain (PRA) protein, which recruits prenylated Rab proteins to their cognate organelles. Among the 52 rice Rab GTPases, OsRab5a interacts with both PIBP4 and PigmR at the ER and PM. Interestingly, the punctate cell periphery localization of PigmR-CC proteins, representing the PigmR microdomains at the PM, is reduced in the *pibp4* and *rab5a* cells, suggesting that PIBP4-OsRab5a module promotes the proper trafficking of PigmR from ER to microdomains at the PM.

The GTPase OsRac1 directly interacts with and is activated by another NLR Pit at the PM to promote Pit-mediated immunity through interacting with ROS generation proteins OsRbohs (Kawano et al. 2010; Nagano et al. 2016). Liang and colleagues found OsRac1 partially co-localizes and also interacts with PigmR at the PM. Notably, PigmR enhances the enzymatic activity of OsRac1 at the PM, indicating that microdomain-localized PigmR promotes blast resistance partially through activating OsRac1 for ROS production. This study sheds new light on the role of NLR microdomain at the PM in mediating blast resistance.

The study of PigmR has undoubtedly substantially advanced our understanding of blast resistance. However, many questions remain unanswered. Are these *PigmR*-mediated defense pathways synergistic or independent? Does PigmR form pentamer at the PM and function as a calcium channel as other CNLs? Is there a cognate AVR protein of PigmR? If any, is this AVR protein recognized by PigmR directly or indirectly? Are there other unexplored functions of PigmR? Given its promising potential in crop improvement, we believe that future research on PigmR will not only deepen our understanding of rice blast resistance but also pave the way for the breeding of new blast resistant rice varieties, and possibly extend beyond rice to other crops.

## Data Availability

Not applicable.

## Abbreviations

PRRs: Pattern recognition receptor
NLR: Nucleotide-binding leucine-rich repeat proteins
PTI: Pattern-triggered immunity
ETI: Effector-triggered immunity
NIL: Near-isogenic lines
*PigmR*: *Pigm*, *Resistant*
*PigmS*: *Pigm*, *Susceptible*
*OsWAK14*: *Wall-Associated Kinase 14*
*OsPAL1*: *Phenylalanine Ammonia Lyase 1*
PIBP1: PigmR-Interacting and Blast resistance Protein 1
PICIs: PigmR-interacting and chitin-induced proteins
OsMETS1: methionine synthase 1
ER: endoplasmic reticulum
PM: plasma membrane
